# Bioinformatics Tools and Benchmarks for Computational Docking and 3D Structure Prediction of RNA-Protein Complexes

**DOI:** 10.3390/genes9090432

**Published:** 2018-08-25

**Authors:** Chandran Nithin, Pritha Ghosh, Janusz M. Bujnicki

**Affiliations:** 1 Laboratory of Bioinformatics and Protein Engineering, International Institute of Molecular and Cell Biology in Warsaw, ul. Ks. Trojdena 4, PL-02-109 Warsaw, Poland; nchandran@genesilico.pl (C.N.); pghosh@genesilico.pl (P.G.); 2 Bioinformatics Laboratory, Institute of Molecular Biology and Biotechnology, Faculty of Biology, Adam Mickiewicz University, ul. Umultowska 89, PL-61-614 Poznan, Poland

**Keywords:** ribonucleoprotein, RNP, macromolecular complex, computational modelling, structural bioinformatics

## Abstract

RNA-protein (RNP) interactions play essential roles in many biological processes, such as regulation of co-transcriptional and post-transcriptional gene expression, RNA splicing, transport, storage and stabilization, as well as protein synthesis. An increasing number of RNP structures would aid in a better understanding of these processes. However, due to the technical difficulties associated with experimental determination of macromolecular structures by high-resolution methods, studies on RNP recognition and complex formation present significant challenges. As an alternative, computational prediction of RNP interactions can be carried out. Structural models obtained by theoretical predictive methods are, in general, less reliable compared to models based on experimental measurements but they can be sufficiently accurate to be used as a basis for to formulating functional hypotheses. In this article, we present an overview of computational methods for 3D structure prediction of RNP complexes. We discuss currently available methods for macromolecular docking and for scoring 3D structural models of RNP complexes in particular. Additionally, we also review benchmarks that have been developed to assess the accuracy of these methods.

## 1. Introduction

Ribonucleic acid (RNA) plays major roles in various biological processes including protein synthesis and gene regulation at the transcriptional and post-transcriptional level. RNA molecules are involved in catalysing biological reactions, controlling gene expression, or sensing and communicating responses to cellular signals [1,2,3]. The majority of the known RNAs exert their function in conjunction with proteins to form RNA-protein (RNP) complexes, at one or more stages of their life cycle. The strength of these complexes can vary from being stable, like in the case of the individual subunits of the ribosome [4], or being able to undergo extensive rearrangements like the spliceosome [5] to being transient, enabling their assembly and disassembly as is observed in the exon junction complex [6]. RNA-protein complexes are involved in many cellular processes, including the maintenance of chromosome ends, transcription, RNA transport and processing, regulation of gene expression, protein synthesis [1,2,3,7], alternative splicing [8], RNA modification and polyadenylation [9,10]. Moreover, the protein non-coding RNAs (ncRNAs) act as scaffolds during macromolecular assembly [11]. For instance, 7SK ncRNA acts as a scaffold for the formation of multiple RNPs and is a major player in the regulation of eukaryotic transcription [12]. Furthermore, RNPs help govern the association of sister chromatid cohesion proteins with genes and enhancers [13]. Defects in RNP interactions are implicated in many diseases ranging from neurological disorders to cancer [14,15]. RNP interactions are thus essential for the critical aspects of cellular metabolism.

RNA-binding proteins (RBPs) often contain structurally and functionally distinct modules. For instance, in all enzymes that act on RNA, RNA-binding is a common feature of catalytic domains that assume various well-defined three-dimensional (3D) structural folds. Some of these domains can bind RNA on their own, while others require dedicated RNA-binding domains (RBDs), which enable the recognition of substrate RNAs [16,17]. Examples of well-studied RBDs include the RNA recognition motif (RRM) [18], the heterogeneous nuclear ribonucleoprotein K homology domain (KH) [19], the double-stranded RNA-binding motif (dsRBM) [20] and the zinc finger domain [21], to name a few. RNA-binding domains are also typical components of proteins involved in the formation of large RNP complexes such as the ribosome or the spliceosome [22,23] and they also occur in proteins that regulate the function of RNAs [24]. Proteins that simultaneously bind multiple sites in RNA often include multiple RBDs [24,25]. Here, we point our readers to a review from the Varani group, on the various RNA-binding strategies of RBPs that exploit the modular nature of RBDs [16]. Apart from RBDs with well-defined 3D structures, RNAs can also be recognized and bound by structurally disordered regions, as in ribosomal proteins, which assume folded conformation only upon binding RNA [26].

Three dimensional structures of RNP interactions have provided important insights into the molecular intricacies governing these interactions, including the specificity of mutual recognition by protein and RNA components and assist in studying the physicochemical principles of RNP interactions. Although 3D structure-derived information is important for understanding biological roles of RNP interactions, experimental determination of RNP complex structures is a slow and laborious process [27,28]. Firstly, many RNA-protein interactions are transient resulting in formation of short-lived complexes. Secondly, there are difficulties associated with the chemical character of the RNA component(s) of the complex. RNA is conformationally more flexible than proteins, and RNA molecules are often structurally heterogeneous. In addition, RNAs are often elongated in shape and in contrast to proteins, exhibit few elements that can stabilize crystal contacts hindering crystal packing. Furthermore, the negatively-charged sugar-phosphate backbone contributes to the repulsion between the molecules. These factors collectively make RNA and RNP structure determination more challenging than protein structure determination.

Until the end of the 20th century there were only a handful of high-resolution structures of RNP complexes available but the number of such structures has exponentially increased over the past decade due to significant improvements in established techniques such as X-ray crystallography [29], as well as the advent of newer technologies like electron microscopy (EM). Several groups developed hybrid techniques and for instance combined the nuclear magnetic resonance (NMR), small angle scattering (SAS), analytical ultracentrifugation (AUC), and/or electron paramagnetic resonance (EPR) experiments [30,31,32]. Despite all these advances, the number of structures for RNP complexes is much lower compared to that of DNA-protein complexes. As of April 2018, 4265 DNA-protein complex structures were available in the Protein Data Bank (PDB). On the other hand, only 2194 macromolecular complexes involving both protein and RNA components (but excluding RNA/DNA hybrids) were available in the PDB. Of these structures, 1642 were solved by X-ray crystallography, 426 by EM, 120 by solution NMR spectroscopy and 6 by other methods such as fibre diffraction. These structures contained 51,101 protein chains interacting with RNAs but many proteins were highly similar to each other. After removing redundant protein chains with sequence identity >90% or >40%, only 2862 or 1302 experimentally-determined RNA-bound proteins respectively, remained.

Over the past decade, various research groups have utilized different tools and techniques to estimate the total number of RBPs in the human proteome. The database of RNA-binding protein specificities (RBPDB) database documents 397 manually curated examples of RBPs from the literature [33]. In 2012, independent reports from the Landthaler and Hentze groups identified 797 and 860 mRNA-binding proteins (mRBPs) in the human proteome, from the HEK293 and the HeLa cells, respectively [34,35] Many of these proteins lack typical RBDs or motifs, or are known to exhibit other functions not related to RNA metabolism [36]. On the one hand, this could indicate that many proteins take part in RNP formation as a part of their life cycle; while on the other hand, such large-scale experimental screens may identify proteins that interact with RNA indirectly, for example, as components of larger protein-protein complexes. The Zhou group used the approach of fold recognition for protein structure prediction and the SPOT-seq technique for binding affinity-based RNA-binding prediction. Using these techniques, they identified 2937 RBPs in humans [37], with a 43.6% coverage of the experimentally reported mRBPs by the Hentze group [35]. More recently, the Tuschl group has consolidated a list of 1542 RBPs in the human genome that have been identified by a combination of bioinformatics approaches and curation of experimental data from literature [38]. Work done by the Sowdhamini group has resulted in the computational prediction of 2625 RBPs in the human proteome [39]. The hRBPome database compares and contrasts the RBPs as reported by the above studies [40]. In 2016, work done by the Preiss, Ørom and Ostareck-Lederer groups have reported 1148 RBPs in cardiomyocytes, 382 RBPs in the nucleus and 402 RBPs in macrophages, respectively [41,42,43]. Obviously, for the majority of these complexes, no structural data is available.

Given the scarcity of experimentally-determined structures of RNP complexes, computational techniques can complement existing data to help elucidation of RNP complex 3D structures. However, while the methodology for prediction and modelling of 3D structures for individual proteins and protein-protein complexes is very well established [44,45,46,47,48,49], there are much fewer methods for predicting and modelling 3D structures of RNA molecules and RNP complexes [50,51,52,53]. In this article, we review computational approaches for modelling of RNP complex structures. We focus on RNP docking, scoring functions and on methods for evaluating the accuracy of predictions, in particular, docking benchmarks and affinity datasets.

## 2. Computational Modelling of RNA-Protein Complex Structures

The prediction of 3D structures of macromolecular complexes is usually done by the computational docking approach, which requires detailed knowledge of structures for all individual components of the complex. Ideally, the docking should be based on the knowledge of high-resolution atomic structures of the components, for example, determined by X-ray crystallography or NMR. However, in many cases, experimentally determined structures of components of the complex are not available. For many applications, these can be substituted by computationally-modelled structures. To this end, a large number of computer programs have been developed, which now allow for reasonably accurate and practically useful predictions of protein 3D structures and reviewing them is beyond the scope of this article. The state-of-the-art in protein 3D structure prediction has been systematically assessed by the Critical Assessment of protein Structure Prediction (CASP) experiment [54]. More recently, the RNA Puzzles experiment has been initiated to assess the state-of-the-art in RNA 3D structure prediction [54,55]. The reader is referred to the most recent articles describing progress in these areas, for example, CASP Round XII [54,55,56] and RNA Puzzles Round III [57].

In computational biomolecular docking, the receptor refers to the larger molecule, while the ligand refers to the smaller one. The docking protocol comprises two steps: (i) conformational sampling, that is, searching for possible conformations and mutual orientations of the docking components that leads to the generation of docked models (called poses or decoys) and (ii) scoring of docked poses: assessing them by a mathematical function that aims to distinguish between models with different degrees of similarity to the unknown “true” structure [58]. Some of the existing docking methods combine both sampling and the scoring steps [59,60,61], while others only specialize in the assessment of docked poses [62,63,64,65].

**Figure 1:**
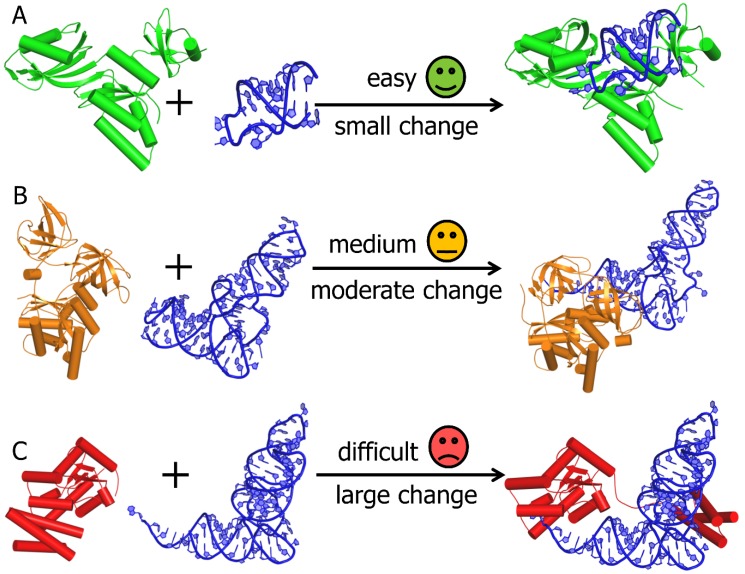
Overview of challenges in RNA-protein (RNP) docking. (A) “easy” docking of tRNA pseudouridine synthase B (1R3F:A) and a small RNA fragment (1K8W:B); the protein undergoes a small conformational change to form the RNP complex (1K8W:A–1K8W:B). (B) “medium difficulty” docking of the Tu elongation factor (1TUI:A) and cysteinyl tRNA (1U0B:A); both components undergo a moderate conformational change to form the RNP complex (1B23:P–1B23:R). For many of the currently available docking tools, it is challenging to model this degree of conformational change. (C) “difficult” docking of l-seryl-tRNA (Sec) kinase (3A4M:A) and selenocysteine tRNA (3ADB:C); the protein undergoes a large conformational change movements to form the RNP complex (3ADB:A–3ADB:C). For most of the currently available docking tools, it is nearly impossible to model such large conformational changes.

A major challenge in computational docking is related to the observation that structures of binding partners often undergo conformational changes during association, in a process known as induced fit. Despite the recent advancement of methods that take macromolecular flexibility into account [66], dealing with conformational changes involving backbone and loop rearrangements still remains the biggest challenge in the modelling of macromolecular complexes [67] (Figure 1). Docking methods differ in the details with which they model conformational changes in the receptor and the ligand. Certain methods model such changes explicitly, making such analyses computationally demanding, whereas the other set of programs focus less on molecular details and introduce a certain level of ‘fuzziness’ [68]. This can be addressed by generating ensembles of conformers. Different research groups have adopted their own strategies towards accomplishing this, such as molecular dynamics (MD) simulations, Monte Carlo (MC) simulations, Normal Mode Analysis (NMA) and use of PDB structure homologs [67].

## 3. RNP Docking Methods (Conformational Sampling with or without Scoring)

Many protein-protein docking methods have been assessed in the Critical Assessment of PRediction of Interactions (CAPRI) experiment, analogous to CASP [69]. In comparison to protein-protein docking, RNP docking has received less attention from computational method developers. However, the number of groups participating in the CAPRI for scoring RNP complexes, as well as the number of available methods for RNP docking, has increased steadily over the years [67,70]. Most of the methods for RNP docking have been developed as modifications to existing protein-protein docking methods, in order to accept nucleic acids as receptors and/or ligands. In order to adapt protein-protein docking methods to RNP docking, the following modifications are necessary: (i) a representation for RNA molecules has to be added to the docking algorithm for data handling purposes (some of the protein-protein docking methods handle only amino acid residues) and (ii) the scoring function has to enable evaluating RNA-protein interactions (some of the protein-protein docking methods can handle non-protein molecules such as RNA but they are unable to take into account RNA-specific interactions). Table 1 lists various RNP docking tools that are freely available as standalone programs or web servers.

**Figure 2:**
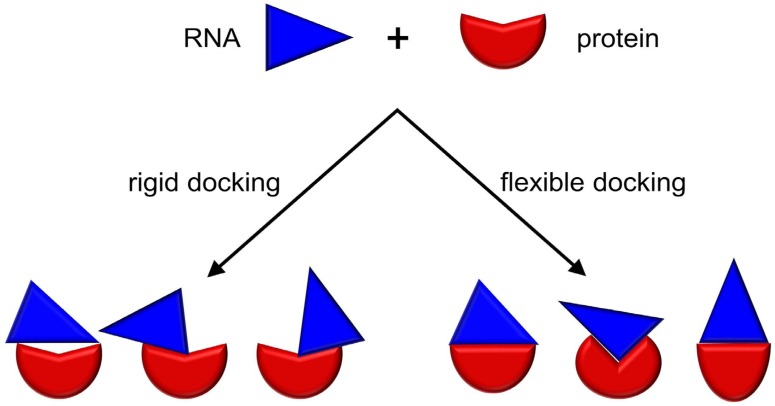
Comparison of rigid and flexible docking methods. A protein and an RNA molecule have been schematically represented as a red and a cyan figure, respectively.

Existing docking methods can be divided into two general classes: (i) rigid and (ii) flexible (Figure 2). Upon RNP complex formation, protein, RNA or both may undergo conformational changes in the backbone (large-scale domain motions and movements in disordered regions) and/or the sidechains. Flexible docking methods attempt to account for these conformational changes in order to predict near-native biological associations. Rigid docking methods do not account explicitly for conformational changes in the structure of the input protein and/or RNA but they may represent these structures in a ‘fuzzy’ way to embody the uncertainty of the bound conformation.

Rigid body docking methods are usually the first method of choice, especially if little is known beyond the structures of the components. By virtue of compromising on computing cost for conformational flexibility, such methods are capable of exploring a bigger search space to identify potential binding sites on the protein and RNA molecules. For example, FTDock developed by the Katchalski-Katzir group generates orthogonal grids to represent the components for docking and then performs a global scan of the search space (translational and rotational) to generate a quick approximate solution of the prediction problem [72]. The results can be, however, only as accurate as allowed by the difference between unbound and bound conformations. In other words, any conformational changes associated with binding contribute to the deterioration of the accuracy of models generated by rigid docking. Thus, apart from the first quick screen of docking solution, rigid docking is recommended only for cases where no major conformational changes are expected to occur.

Flexible docking can simulate conformational changes; however, its utility is also correlated with the degree of the structural change between the unbound and bound forms. It is a method of choice for cases in which the conformational search space is relatively small or for the purpose of local structure refinement. In fact, none of the typical docking methods can reliably predict the structures of complexes that undergo large and complex conformational changes during binding—for this, multi-scale methods are a recommended solution (see below). An example of a popular flexible docking method is HADDOCK, developed by the Bonvin group. It can take the information from user-defined restraints (for example, from experimental data) to drive the docking [86], as well as take into account the flexibility of both the protein and the nucleic acid [87]. Unlike many other methods, HADDOCK can use the nucleic acid both as a receptor, as well as a ligand. A user-defined set of residues are allowed to be conformationally flexible during the docking process. Thus, HADDOCK is capable of performing well in cases where there are local rearrangements in and around these residues. The program is available as a web server [61] and also as a standalone tool [82]. HDOCK, developed by Huang group, differs from the other existing methods, in being able to accept both protein structures and sequences as input [83]. However, it can only accept input RNA structures (and not sequences) for the docking protocol. In case of a protein sequence input, HDOCK performs a sequence similarity search against the PDB using the HHSuite package [88] to identify a homologous structure, which is then used as the template for a subsequent MODELLER-based modelling step [89]. The theoretical model of the protein structure is then used for the docking step.

## 4. Other Methods for Three Dimensional Structure Prediction of RNP Complexes

The 3D structures for RNP complexes can be modelled by methods that do not involve explicit docking, or in which the docking is only a minor component of calculations.

Template-based modelling of complexes (in which the structure of one complex is modelled based on the structure of a related complex used as a template) is widely used for protein-protein interactions [90] but less investigated for the modelling of RNP complexes. Protein RNA Interaction ModEling (PRIME) [91] is one such method for predicting RNP complex structures, based on structurally similar complexes with experimentally determined structures. The structural similarity of the individual components to the those in the “template” structure is important to ascertain the binding mode with certainty. It performs well in cases where “free” docking fails (demonstrated by the authors by testing the method on the RNP docking benchmark set of the Fernandez-Recio group) and is capable of accounting for conformational changes upon binding [91]. RStrucFam, from the Sowdhamini group, is a method for predicting cognate RNA partners for RBPs, based on their sequence similarities with the protein components of known RNP complex structures [92].

Another strategy in the prediction of RNP complex structures is MD simulations [93]. The protein and RNA can be driven to bind by placing them in appropriate force fields with restraints but in general, MD is incapable of accurately simulating binding events and large conformational changes that occur on time-scales larger than microseconds [94]. On the other hand, near-native docked poses generated by macromolecular docking can be used as starting structures for MD, allowing the system to readjust to the minimal energy. Moreover, including MD force fields in scoring can improve docking [95]. Work done by the Kameda group in 2016 demonstrated an improved accuracy for RNP complex prediction by a rigid protein-protein docking method [95], ZDOCK, which originally was not parameterized for RNA. The following modifications in the ZDOCK method were introduced by the authors: (i) derivation of the physicochemical properties of nucleic acids (such as partial charge of atoms, Van der Waals radii etc.) from MD simulation force fields for nucleic acids (such as, AMBER and CHARMM) and (ii) introduction of electrostatic interactions into the scoring function based on the AMBER94 force field. The Wang group applied a combination of docking and MD followed by binding energy calculations to identify the binding mode of RNA to carbon storage regulator A protein (CsrA), which was previously unknown [96]. The authors calculated binding free energies using molecular mechanics combined with the generalized Born and surface area continuum solvation (MM/GBSA) method [97]. This study has identified the binding pockets in CsrA that could be targeted by small molecules to prevent RNA binding, without interacting with the RNA [96].

Another class of methods have been developed to model multi-scale resolution models of large macromolecular complexes. These methods often represent components as rigid bodies or as flexible forms, depending on the availability of information about their structure and dynamics. They usually rely on additional information encoded in the form of spatial restraints to define interactions between the components. The M3 framework from the Carlomagno group uses sparse and hybrid experimental data to model structures of macromolecular complexes, starting from 3D structures of individual domains, monomers and subcomplexes. These components can undergo rearrangements and yet retain their overall 3D structure upon complex formation [98]. The experimental data are used to derive interatomic distances and/or molecular shapes, to guide the modelling process. The Integrative Modelling Platform (IMP) software package from the Sali group [99] is an example of a hybrid method. It performs modelling of simple biomolecules, as well as large macromolecular assemblies, by integrating data from various experiments. The preparation of the input data for IMP requires advanced knowledge of the system analysed. Recently, our group developed PyRy3D (http://genesilico.pl/pyry3d/), a multiscale modelling method that enables the construction of models for very large macromolecular complexes with components of known or unknown 3D structure. PyRy3D aims at cases of data-driven modelling of complexes where the users have only limited knowledge about the system analysed and wish to test alternative hypotheses. PyRy3D applies a MC search to sample the space of solutions restricted by various spatial restraints that determine 3D shapes or interactions between subunits of complexes. It has been used to model RNPs such as the complex between the 2′-5′-oligoadenylate synthetase OAS1 and the 3′-terminal region of the West Nile Virus (WNV) RNA genome [100].

## 5. Standalone Scoring Methods for RNA-Protein Complexes

For the modelling of RNP complex structures, it is often useful to consider predictions from different modelling methods, followed by a ranking of the alternative poses using external scores. Scoring functions are essential for distinguishing between models of various accuracy, in particular, to discriminate between models that are close enough to the “true” structure to provide useful functional information and models that are inaccurate and could lead to wrong conclusions. Standalone scoring methods are invaluable for comparison of models obtained with different modelling approaches that rely on different internal scoring functions. The scoring of 3D structural models may be based on the following considerations: (i) verification to what extent the given decoys agree with independently obtained information (for example, with experimental data or with independent computational predictions) and (ii) evaluation of the overall quality of fitting between the protein and RNA structures.

For scoring of models with an independent dataset, a large number of approaches exist. A variety of experimental methods can be applied to study RNA, protein and RNA-protein complex structures, which can be translated into spatial restraints. Furthermore, numerous computational methods are available for predicting RNA-binding residues in proteins [101,102]. Once the models of RNP complexes become available, scoring based on external information (including experimental data) can be performed with various computational tools. As an example, FILTREST3D [103] allows scoring of decoys based on a combination of distance restraints with other factors such as local or global structure or molecular shape. It also allows the use of logical operators to enable sets of alternative restraints. pyDockRST also uses the percentage of satisfied distance restraints derived from experimental data, along with electrostatics and desolvation binding energy, to score and rank docking poses [104].

In the absence of independent data, structural models of RNP complexes can be evaluated with generic scoring functions, which can be broadly categorized into three types: force field, empirical and knowledge-based. Force fields are the functional form and parameter sets used to calculate the potential energy of a system of atoms or coarse-grained points. Empirical scoring functions are based on counting the number of various types of interactions at the interface of RNP complexes. Knowledge-based scoring functions, also known as statistical potentials are mathematical functions derived based on statistical observations of interactions at the interface of known RNP complexes. A list of methods available for computational scoring of RNP complexes is presented in Table 2.

The Varani group was one of the first groups to development scoring functions, for RNA-protein interactions. Initially, they developed a statistical potential based on hydrogen bonding (H-bond) at the RNP interfaces [105]. However, H-bonds represent only a portion of various types of interactions occurring at the RNP interfaces [110,111]. Later, the same group developed an all-atom, distance-dependent statistical potential for predicting sequence-specific recognition between RNA and protein [106]. The Varani’s all-atom potential treats the interactions between chemically similar atoms (based on the CHARMM atom definition) in the same way and as a result, it contains only a distance-dependent multiple bin term. A coarse-grained, distance-dependent pairwise residue-ribonucleotide propensity was derived by Fernandez-Recio group to score the docking poses [107]. In their approach, the entire residue is represented as a single interaction centre and therefore it uses a single bin (i.e., the presence or absence of interaction) to calculate the potential. Zhou and co-workers developed dRNA [108]; a volume-fraction corrected distance-scaled, finite ideal-gas reference (DFIRE) energy function for RNP interactions [112]. However, the dRNA method requires known RNP complex structures as templates and has limited applications when the RBPs used have novel binding modes different from those in the template structures. Our group developed two knowledge-based potentials: the quasi-chemical potential (QUASI-RNP) and the Decoys As the Reference State potential (DARS-RNP) [62]. These statistical potentials use a coarse-grained representation, which ignores molecular details and hence is insensitive to minor conformational inaccuracies. The reference states used in these two potentials differ. While QUASI-RNP uses mole fractions of residues as a reference state, DARS-RNP uses decoys. Both these potentials have the same mathematical base except for the references states and use multiple bins for distances as well as orientations.

The Zacharias group developed a distance-dependent, coarse-grained force field for RNP interactions [109]. During a testing of this potential, the authors observed during the testing of this potential that it allowed moderate conformational changes. The potential allows protein-RNA docking followed by energy minimization in the rotational and translational degrees of freedom of the binding partners [108] Wang and co-workers developed four different pairwise residue-nucleotide potentials. These potentials were derived from the pairwise residue-nucleotide propensities with or without considering the protein and/or RNA secondary structural elements [63]. The authors concluded that the RNA secondary structure information contributed more significantly than the protein secondary structure in discriminating the correct 3D structures of RNP complexes.

The Xiao group developed a scoring function, Distance- and Environment-dependent Coarse-grained and Knowledge-based potential for RNP complexes (DECK-RP), to evaluate the docked poses generated by RPDOCK [64]. DECK-RP combined the advantages of both Wang’s potential [63] and DARS-RNP [62]. The secondary structure context was considered for calculating the pairwise propensities. DECK-RP uses a reference state, which includes a decoy-based component and a mol-fraction corrected component. The Zou group developed a scoring function, ITScore-PR, based on the atomic distance-dependent potentials derived from known RNP complex structures, which uses a physics-based iterative algorithm [65]. The authors demonstrated that many algorithms performed better for rigid body docking in which the components were derived from bound structure rather than for flexible docking where the components were derived from the unbound structures. The Xiao group developed a new knowledge-based potential, RPRANK. The conformation differences between residue-base pairs between standard pairs from native structures and decoys were used to calculate the statistical potential [71]. 

The approximate utility of various potentials, with respect to the accuracy of docking decoys analysed, is indicated as a decoy discrimination threshold in Table 2. For instance, some of the fine-grained or high-resolution (all-atom) methods are useful for discriminating decoys that exhibit root mean square deviation (RMSD) to the reference structure <3 or <5 Å (decoys that are less accurate exhibit essentially random score, not related to the accuracy) and some coarse-grained or low-resolution methods can discriminate decoys up to ~10–15 Å from the reference structure but they are usually not appropriate for discriminating between decoys that are very close to the native structure.

The availability of different docking methods and scoring functions allows for various combinations to be applied. Matching the docking procedure with a scoring function of a similar resolution is recommended. For example, we developed NPDock, a web server for low-resolution RNP structure prediction [60], which first performs rigid body RNP docking using GRAMM [73] and then scores the decoys with different coarse-grained statistical potentials QUASI-RNP and DARS-RNP, also developed by our group [62]. Likewise, pyDockWEB generates rigid-body docking orientations by FTDock and evaluates them by the pyDock scoring function [78]. On the other hand, high-resolution potentials that operate at the atomic level (e.g., the ones developed by the Varani group) are expected to work well only with docking poses that are expected to be very close to the native structure, which often can be generated only by the flexible docking methods that allow for precise modelling of atomic interactions.

An important consideration in the selection of decoys is not only scoring individual models but also finding ensembles of similar models [113]. In general, an ensemble of structurally similar models with very good scores is often indicative of a more accurate prediction than a single model with the best score. However, several clusters of solutions with similar scores may exist. Hence, docking methods often report not just one best-scored model but representatives of several largest clusters (usually up to three or five). Various clustering strategies exist and, so far, no comprehensive study was performed to identify the best approach that could work with decoy datasets generated with different docking methods and scored with different functions. The strategy adopted in our method NPDock is based on grouping of decoys with low RMSD values. First, an RMSD matrix is generated for all pairs of decoys. Second, the structures with RMSD values below a specified cut-off value are grouped into one cluster, which is removed from the dataset and the process is iterated. The program reports the medoids of the biggest three clusters as well as the model with the overall best score [62].

## 6. RNA-Protein Three-Dimensional Structure Datasets for Benchmarking the Computational Docking Methods and Their Applications

**Figure 3:**
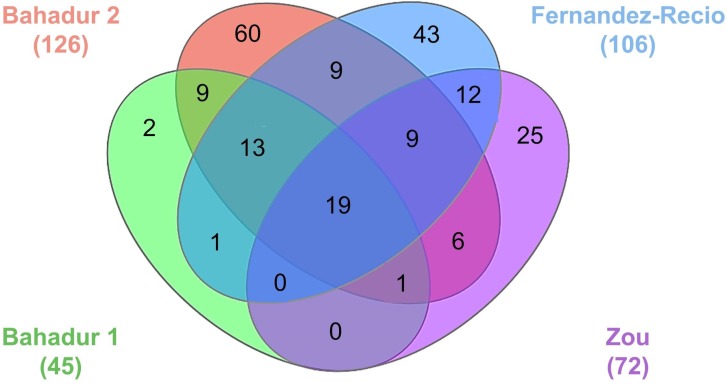
The relationship between RNP docking benchmarks datasets. Nineteen RNP structures are common to all four benchmarks. Two hundred nine RNP structures are represented by all four benchmarks together.

Evaluating the performance of existing docking and scoring methods requires datasets of 3D atomic coordinates of RNP complexes that serve as references (“true” structures) for validating the predictions. In this section, we discuss the various benchmark datasets curated by different groups (Table 3, Figure 3). The different benchmarks employ different criteria for selecting the complex structures and the corresponding unbound structures. The first benchmark dataset for RNP docking was assembled by the Bahadur group, which only included structures determined by X-ray crystallography [114]. This benchmark consists of 45 non-redundant RNP complexes and their corresponding unbound structures. There are nine unbound-unbound test cases for which both the protein and RNA are available in an unbound form and 36 unbound-bound test cases for which only protein is available in unbound form (Table 3). This benchmark is divided into four structural classes: (A) complexes with tRNA (16 cases), (B) complexes with ribosomal proteins (three cases), (C) complexes with duplex RNA (10 cases) and (D) complexes with single-stranded RNA (16 cases). In addition, this benchmark divides the dataset into three categories based on the conformational changes undergone by the interface Cα atoms: (R) rigid body (i-rmsd_Cα_ < 1.5 Å) with 34 cases, (S) semi flexible (1.5 Å ≤ i-rmsd_Cα_ ≤ 3.0 Å) with eight cases and (X) full flexible (i-rmsd_Cα_ ≥ 3.0 Å) with three cases. Here, i-rmsd_Cα_ is defined as the RMSD of the interface Cα atoms after superposing the bound and unbound structures. The authors have calculated the interface residues using the PRince webserver [115]. The benchmark from the Bahadur group was later updated with 126 non-redundant RNP complexes [116]. This includes 21 unbound-unbound cases, 95 unbound-bound cases and 10 are bound-unbound cases. Of the 21 unbound –unbound types, 12 are pseudo-unbound where the RNAs are taken from a different RNP complex. The dataset is divided into four structural classes with 28, 5, 40 and 53 cases in classes A, B, C and D, respectively. The current version of this benchmark consists of 72, 25 and 19 cases in R, S and X categories.

An extended benchmark including both experimental structures and homology models was curated by the Fernandez-Recio group with 106 test cases. In this benchmark dataset, 71 out of 106 entries were taken from crystallography or NMR experiments, while 35 entries were built using homology modelling [117]. Of the experimental structures available, there are nine unbound-unbound cases and 62 unbound-bound forms. Among the nine unbound-unbound cases, four cases are pseudo-unbound where the RNAs are taken from a different RNP complex. The homology-modelled cases in this benchmark consist of five unbound–model, eight model-unbound, 19 model-bound and three model-model protein-RNA cases. The benchmark is divided into three categories for docking predictions based on the conformational changes undergone by the Cα and P atoms at the interface of RNP complexes: easy (i-rmsd_Cα+P_ ≤ 2.5 Å) with 64 cases, intermediate (2.5 Å ≤ i-rmsd_Cα+P_ ≤ 5.0 Å) with 24 cases and difficult (i-rmsd_Cα+P_ > 5 Å) with 18 cases.

The Zou group developed another docking benchmark for RNP complexes with 72 cases, of which, 52 are unbound-unbound cases, 17 are unbound-bound cases and three are bound-unbound cases [118]. In bound-unbound cases, only RNA structures are available in an unbound form. Based on the conformational changes undergone by the interface Cα and C4′ atoms and the fraction of native contacts this dataset is classified into three categories: easy (i-rmsd_Cα+C4′_ ≤ 1.5 Å or f_nat_ ≥ 0.8) with 49 cases, medium (1.5 Å < i-rmsd_Cα+C4′_ ≤ 4.0 Å and 0.4 ≤ f_nat_ < 0.8) with 16 cases and difficult (i-rmsd_Cα+C4′_ > 4.0 Å or f_nat_ < 0.4) with seven cases. 

The docking benchmarks discussed in this section were widely used, i.e., to test docking methods [79,83], to develop knowledge-based scoring functions for studying RNP interactions [65,119,120], to predict RNA-binding sites in proteins [115,121,122], to investigate the role of water molecules [123], to find the binding hot-spots and to predict binding affinities [124]. Besides, the availability of bound and unbound structures assists in the development of new physicochemical and structural parameters for quantifying the changes occurring in RNA-protein interaction sites upon binding [110,125,126,127].

A number of studies covering the general properties of RNP complex structures used the benchmark dataset developed by the Bahadur group, i.e., due to the stringent criteria on the resolution (better than 3.0 Å) and redundancy (sequence identity ≤ 35%). Studies of RNA-protein binding sites of complexes in this dataset included the analysis of the role of water molecules in RNP complex formation [123], the study of sequence conservation at RNP interfaces [124] and the quantification of solvent accessibility at interfaces of RNP complexes [125]. The Zou group benchmark used a more relaxed resolution cut-off of 4.0 Å compared to other docking benchmarks. It included more low-resolution structures, which may not be suitable for the development of high-resolution scoring functions or evaluation of physicochemical parameters. However, the low-resolution structures may be useful as templates for comparative modelling or for development of coarse-grained methods. The benchmark developed by Fernandez-Recio group went even further, beyond pairs of bound and unbound structures determined experimentally for the same protein and RNA molecules, to include structures obtained by homology modelling in cases, where experimental structural data existed only for related components [117]. This dataset allows to address some challenging problems, for which low-resolution structures are sufficient. However, caution must be applied while using these structures, as errors in structures obtained by modelling may affect the conclusions.

## 7. Datasets for Ribonucleic Acid-Protein Binding Affinity Prediction and Their Applications

RNA-Protein interactions are often affinity-driven processes, where the specificity of binding is determined by the conformation adopted by the molecule to bind with its partner. The free energy required for RNA and/or protein molecules, to adopt the particular conformation required for binding, is a determining factor in such binding events.

The first RNP affinity benchmark was developed in 2013 by the Liu group with 73 cases [128]. A comprehensive and up-to-date affinity benchmark for RNP complexes is still missing. A dataset of affinity values for alanine substitutions in protein components of RNP complexes was curated by Bahadur group for 14 RNP complexes [124]. The changes in affinity values upon alanine substitutions is indicative of the role of the corresponding residue in the binding process. The knowledge of affinity values helps in defining the active residues for guided docking of RNP complexes. The dataset reports 94 experimental affinity values for 14 native structures and 80 variants bearing single residue alanine substitutions [124]. This dataset was later expanded by the Deng group to include 49 RNP complexes [129]. The dataset reports 334 experimental affinity values for 49 native structures, 254 variants with alanine substitutions and 31 other substitutions. The dbAMEPNI dataset curated by Mitchell and Zhu groups reports affinity data for 51 RNP complexes [130]. The dataset includes experimental affinity values for 193 alanine substitutions in RNPs.

It is important to draw our readers’ attention to the fact that the bioinformatics methods discussed in this review are for predicting the possibilities in which a given protein and an RNA interact with each other and they assume that their interaction does happen. These methods are not appropriate for ascertaining if the protein and the RNA interact or not, or for predicting which RNA binds to a given protein or vice versa. Predicting the binding affinity would help us determine whether the molecules bind to each other. Methods for computational prediction of binding affinities are however still in their infancy. They require high-quality datasets of experimentally determined affinity values, which are currently sparse. The affinity dataset curated by the Bahadur group was used in the development of an algorithm to predict hot-spot residues within RNA-binding sites and is available as a web server HotSPRing [124]. The same dataset was used by Pires and Ascher to develop the mCSM-NA web server, which uses graph-based signatures to predict the impact of single residue substitutions on nucleic acid binding affinity [131]. The Deng group have developed PrabHot, another method to predict the hot spot residues at RNA-protein interfaces [129].

## 8. Conclusions

Over the past decade, there has been a growing interest in investigating RNP interactions. This is apparent from the increasing number of structures of RNP complexes that have been deposited in the PDB database per year (197 in 2017 vs. 87 in 2007), as well as a greater number of publications that appear each year in the PubMed database, with the keyword “RNA-binding proteins” (1405 in 2017 vs. 1278 in 2007). However, computational methods for prediction of RNP complex structures using information from structures of the individual components or directly from sequences, have been sought after due to the difficulties associated with the experimental determination of their structures.

Computational prediction of RNP 3D structures can provide important information in cases where standard approaches for experimental structure determination fail. In our own work, we encountered numerous RNP systems, for which we or our collaborators attempted to crystallize the complex but failed to obtain diffraction-quality crystals. Consequently, the available data (in some cases including the crystal structure of the protein partner in the apo form) were used to guide macromolecular docking and modelling of RNA-protein interactions, providing functional insight that could not be obtained from structures of the components in isolation from each other. One example includes the BsMiniIII endonuclease, which crystallized only in the apo form but not in complex with its dsRNA substrate and for which an RNP complex structure was modelled [132] and used to guide the successful engineering of substrate preference [133]. For CMTr2 methyltransferase, we were unable to obtain sufficient amounts of protein for crystallization and had to model the structure of the complex with a 5′-capped RNA substrate to obtain insights into the mechanism of substrate recognition [134]. The structure of the archaeal tRNA methyltransferase Trm10 was obtained only in the RNA-free form and the protein-tRNA complex had to be modelled, with additional experimental data as restraints [135]. Finally, for several bacterial rRNA methyltransferases including ErmC’ [136] RlmH [137] and NpmA [138] only the protein structure was known and while the ribosome structure was known, the determination of the RNA-protein (or protein-ribosome) complex structure has proven unsuccessful, hence the structural insight into their mechanism of action had to be obtained by docking/modelling.

The approaches for modelling RNP complexes discussed in this paper, though capable of providing practically useful predictions, suffer from various limitations. One of the biggest drawbacks concerning RNA modelling is the relative scarcity of experimentally determined RNA and RNP complex structures that can be used for training and testing the methods as well as templates in comparative modelling approaches. The value of RNP structures for the community of computational biologists can be illustrated by the repeated calls from organizers of the CAPRI and RNA Puzzles initiatives to experimental researchers for proposing the newly determined structures as prediction targets. One can hope that the significance of this problem will wane with the growing interest in RNA structural biology, leading to an increase in the availability of solved structures solved each year.

**Figure 4:**
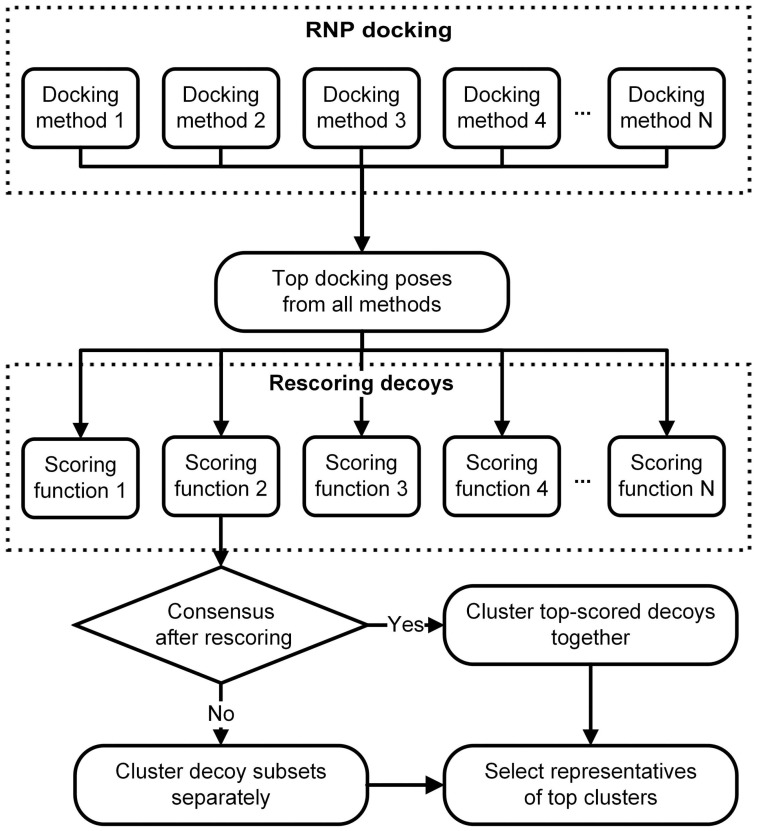
Schematic representation of the workflow for RNP docking. The docking strategy presented here combines the strengths of several docking and scoring methods.

In order to obtain biologically, chemically and physically relevant predictions, it may be advantageous to combine various existing methods for docking and scoring. Such meta-prediction was successfully applied in structural bioinformatics for modelling protein structures [139] and for protein-protein docking [140]. Thus, RNP docking can be performed using different methods and the top scored docking poses from each of these studies can be selected. The top decoys can be then re-scored using various scoring functions. The top scored decoys from the various scoring experiments can be chosen for further analysis, for example, clustering. If the scoring methods reach consensus, then poses obtained from different methods can be clustered together. In the absence of a consensus scoring, top models proposed by different methods can be suggested as alternative solutions. The proposed RNP meta-docking workflow is represented schematically in Figure 4.

One of the biggest challenges in docking (and in particular in RNA-protein docking) is the molecular flexibility [125] and the computational complexity associated with flexible docking. The current methods for scoring models of RNP complexes are quite accurate for evaluating poses generated by rigid docking methods. However, generating conformations that are closer to the bound conformation than the starting unbound structure and discriminating these conformations from all the others is a daunting task for computational methods. The existing computational docking algorithms seldom take into account conformational changes that may occur upon binding of the ligand to the receptor, in RNA and/or protein component(s). Similarly, the available scoring methods also have limited discriminative power to identify near-native structures when the binding protein and/or RNA undergoes large conformational changes. A possible solution to this issue is to combine the existing tools that enable template-free modelling of the protein and RNA components, with scoring functions for the assessment of intermolecular contacts. As a first step towards this approach, modelling techniques have been developed that accept models obtained by for example, rigid body docking of “unbound” protein and RNA structures as input and perform only local refolding of protein and RNA molecules directly involved in interactions.

The conceptual similarity of successful algorithms for structural modelling of protein and RNA 3D structures [141] suggests the feasibility of combining them into unified modelling methods. Combining theoretical predictive methods with low-resolution experimental analyses is also expected to provide synergy to such attempts. Recently, it was demonstrated that the structures of many large RNP complexes, such as the spliceosome, may be modelled using cryo-EM maps as molecular envelopes. The structures of components could be fitted into such envelopes, using restraints from biochemical experiments and other bioinformatics-based predictions [142]. In order to achieve this, new multi-resolution modelling methods and new ways of encoding experimental data are required [143]. We hope that the recent surge of interest in studying RNP interactions will encourage both biologists and software developers alike, to use bioinformatics tools for obtaining structural insights into the biological systems, guided by available experimental data, as well as in proposing and developing new algorithms and their user-friendly implementations.

## Figures and Tables

**Table 1:** Comparison of existing RNP docking methods. The majority of these methods are modified from existing protein-protein docking methods. The type of docking algorithm (rigid or flexible) and their availability (web server and/or standalone) are indicated.

Name	Modified from Protein-Protein Docking Method	Docking Method (Rigid/Flexible)	Availability		References
			Web Server	Standalone	
3dRPC	✗	Rigid	✓	✓	[64,71]
ClusPro	✓	Rigid	✓	✗	[59]
FTDock	✓	Rigid	✗	✓	[72]
GRAMM	✓	Rigid	✓	✓	[73]
Hex	✓	Rigid	✓	✓	[74]
ICM	✓	Rigid	✗	✓	[75]
NPDock	✗	Rigid	✓	✗	[60]
PatchDock	✓	Rigid	✓	✓	[76]
PEPSI-DOCK	✓	Rigid	✗	✓	[77]
pyDock	✓	Rigid	✓	✓	[78]
RosettaDock	✓	Rigid	✓	✓	[79]
ZDOCK	✓	Rigid	✓	✓	[80]
ATTRACT	✓	Flexible	✓	✓	[81]
HADDOCK	✓	Flexible	✓	✓	[61,82]
HDOCK	✗	Flexible	✓	✗	[83]
PIPER	✓	Flexible	✗	✓	[84]
Prime	✓	Flexible	✗	✓	[85]

**Table 2:** List of scoring methods for RNP docking. The representation of the molecules (all-atom or coarse-grained), the type of statistical function and the availability of these methods (web server and/or standalone) have been listed in this table.

Name	Structure Representation	Scoring Method	Decoy Discrimination Threshold (RMSD)	Availability as a Standalone Tool	Reference
Varani’s H-bonding potential	All-atom	H-bonding potential	<3 Å	✗	[105]
Varani’s all-atom potential	All-atom	All-atom distance-dependent	<5 Å	✗	[106]
Fernandez’s potential	Coarse-grained	Pairwise residue-ribonucleotide propensity	<10 Å	✗	[107]
dRNA	All-atom	Volume-fraction corrected DFIRE energy function	NA *	✗	[108]
DARS-RNP and QUASI-RNP	Coarse-grained	Quasi-chemical potential and decoys as the reference state potentials	<10–15 Å	✓	[62]
Zacharias’ potential	Coarse-grained	Distance-dependent, coarse-grained force field for protein–RNA interactions.	<8 Å	✗	[109]
Wang’s potentials	Coarse-grained	Pairwise residue-ribonucleotide propensity with secondary structure information	<10 Å	✗	[63]
Deck-RP	Coarse-grained	Distance and environment dependent	<15 Å	✓	[64]
ITScore-PR	All-atom	Pairwise distance dependent atomic interaction potential	<10 Å	✓	[65]
RPRANK	Coarse-grained	Pairwise residue-nucleotide RMSD	< 10 Å	✓	[71]

* data not available

**Table 3:** List of RNP docking benchmarks. The number of unbound-unbound, unbound-bound and bound-unbound test cases is listed in this table.

Benchmark	Number of Test Cases	Unbound-Unbound	Unbound-Bound	Bound-Unbound	References
Bahadur group 1	45	36	9	0	[114]
Bahadur group 2	126	95	21	10	[116]
Fernandez-Recio group	106	81	25	0	[117]
Zou group	72	52	17	3	[118]
